# gAChR antibodies in children and adolescents with acquired autoimmune dysautonomia in Japan

**DOI:** 10.1002/acn3.51317

**Published:** 2021-02-23

**Authors:** Makoto Yamakawa, Mari Watari, Ken‐Ichi Torii, Ichiro Kuki, Masashi Miharu, Momoko Kawazu, Akihiro Mukaino, Osamu Higuchi, Yasuhiro Maeda, Tokunori Ikeda, Koutaro Takamatsu, Nozomu Tawara, Keiichi Nakahara, Hidenori Matsuo, Mitsuharu Ueda, Takao Takahashi, Shunya Nakane

**Affiliations:** ^1^ Department of Neurology Graduate School of Medical Sciences Kumamoto University Kumamoto Japan; ^2^ Department of Pediatrics Tokyo Metropolitan Ohtsuka Hospital Tokyo Japan; ^3^ Department of Pediatric Neurology Osaka City General Hospital Osaka Japan; ^4^ Department of Pediatrics National Hospital Organization Tokyo Medical Center Tokyo Japan; ^5^ Department of Molecular Neurology and Therapeutics Kumamoto University Hospital Kumamoto Japan; ^6^ Department of Clinical Research National Hospital Organization Nagasaki Kawatana Medical Center Nagasaki Japan; ^7^ Department of Neuroimmunology Nagasaki University Graduate School of Biomedical Sciences Nagasaki Japan; ^8^ Department of Neurology National Hospital Organization Nagasaki Kawatana Medical Center Nagasaki Japan; ^9^ Department of Clinical Investigation (Biostatistics) Kumamoto University Hospital Kumamoto Japan; ^10^ Department of Pediatrics Keio University School of Medicine Tokyo Japan

## Abstract

**Objective:**

Patients with acquired autonomic dysfunction may have antibodies specific to the ganglionic nicotinic acetylcholine receptor (gAChR). However, the clinical features of children and adolescents with acquired autonomic dysfunction (AAD) remain unclear. This study aimed to determine the clinical features of pediatric patients with acquired autonomic dysfunction.

**Methods:**

This study retrospectively examined a series of patients of AAD with serum gAChR antibodies who were referred to our laboratory for antibody testing between January 2012 and April 2019. The study included 200 patients (<20 years, 20 cases; ≥20 years, 175 cases) with clinical features of AAD.

**Results:**

Upon comparing pediatric and adult patients, we found that antecedent infection and autonomic symptoms at onset with gastrointestinal symptoms occurred more frequently in children with AAD. We confirmed that four children (20.0%) met the diagnostic criteria for postural orthostatic tachycardia syndrome (POTS). A significantly higher number of children than adults had POTS (*P* = 0.002). In addition, upper GI dysfunction was more prevalent in children than in adults (*P* = 0.042). In particular, nausea and vomiting occurred in 60.0% of children with AAD and in 21.1% of adults (*P* < 0.001). The frequency of paralytic ileus was significantly higher in children with AAD (20.0%) relative to adults (6.3%) (*P* = 0.030). Regarding extra‐autonomic manifestations, encephalopathy was more frequent in children (15.0%) than in adults (1.1%) (*P* < 0.001).

**Interpretation:**

Pediatric AAD patients have their own clinical characteristics, and these features may be unique to children and adolescents.

## Introduction

Autoimmune autonomic ganglionopathy (AAG) is a rare disorder characterized by autonomic failure associated with the presence of antibodies (Abs) specific to the ganglionic nicotinic acetylcholine receptor (gAChR).^1^, ^2^, ^3^ Furthermore, autoimmune GI dysmotility (AGID) has recently been recognized as a limited form of autoimmune dysautonomia.^4^ In 1993, Schondorf and Low suggested that idiopathic postural orthostatic tachycardia syndrome (POTS) could be a possible manifestation of a mild form of autonomic neuropathy.^5^ Several groups previously confirmed these autonomic manifestations and identified complications in AGID or autoimmune POTS patients who were seropositive for the gAChR Abs.^4^, ^6^, ^7^, ^8^ AGID, autoimmune POTS, and AAG are included in the clinical entity characterized by acquired autoimmune dysautonomia (AAD).

Differences and similarities in the clinical features of neuroimmunological disorders between adults and children have been a focus of research in recent years.^9^, ^10^ For example, children with multiple sclerosis are more likely to clinically show a polysymptomatic onset.^11^ Several studies have demonstrated that, in demyelinating diseases during childhood, Abs against the myelin oligodendrocyte glycoprotein are present in high titers, in particular in younger children.^12^, ^13^ Though there have been several case reports on AAG thus far, these reports included both gAChR Abs‐positive and negative cases.^14^, ^15^, ^16^, ^17^, ^18^ Our laboratory previously established a luciferase‐reporter immunoprecipitation system (LIPS) assay for detecting gAChR Abs,^3^, ^19^ but the prevalence and clinical features of children and adolescents with AAD remain unclear. To address these questions, this study compared the clinical characteristics of AAD between children and adults, seeking to determine the clinical features of pediatric AAD in gAChR Abs‐positive Japanese patients.

## Methods

### Ethical approval

All subjects provided written informed consent to participate in this study. Ethical approval was granted by the Ethics Committees of Nagasaki Kawatana Medical Center (approval number 2011–21) and the Kumamoto University Hospital (approval number 1281).

### Study design and participants

**Figure 1:**
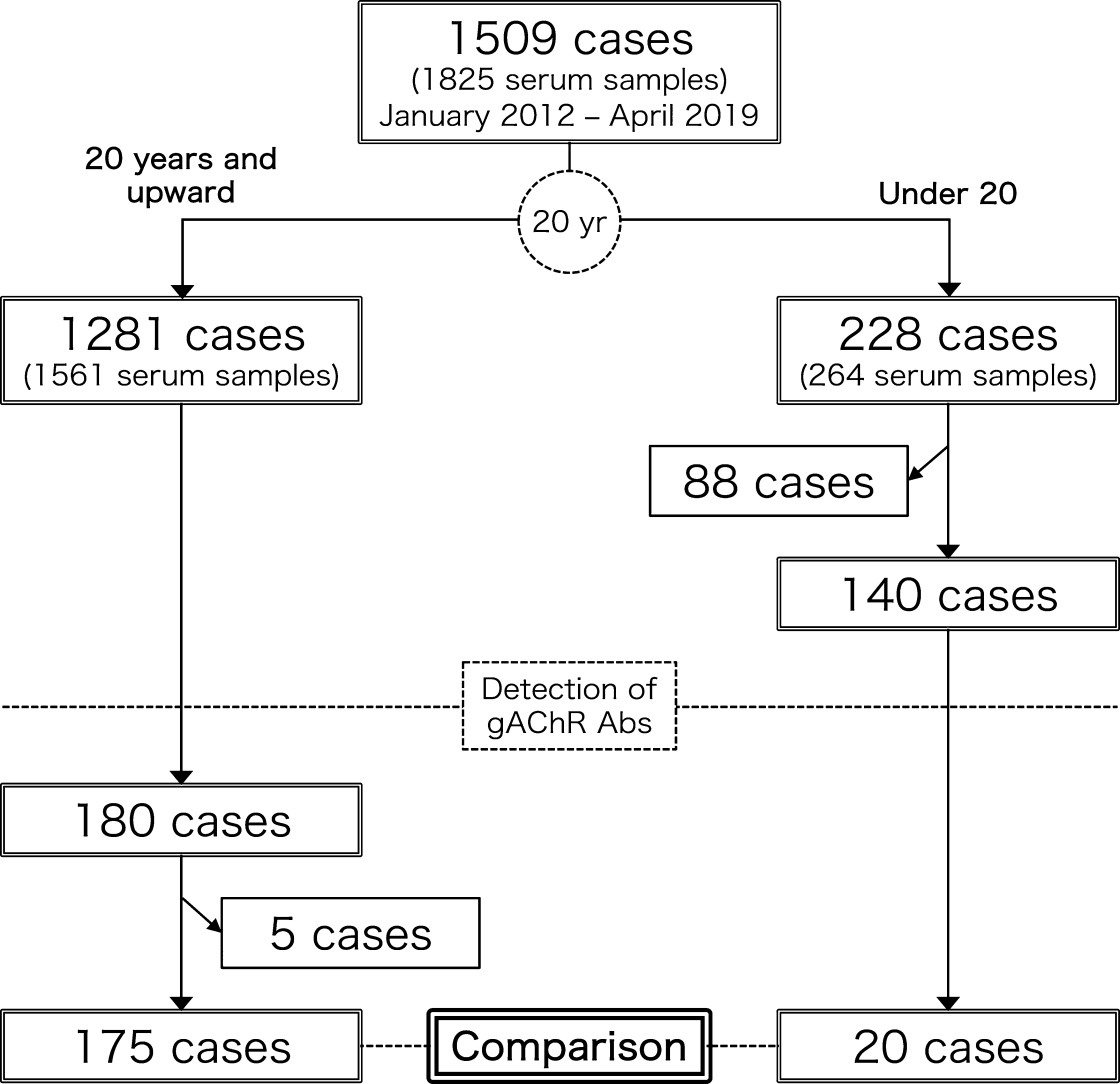
Study recruitment flow chart. We obtained 1,825 serum samples (from 1,501 patients) from teaching and general hospitals throughout Japan between January 27, 2012 and April 2, 2019. We divided the enrolled patients into two groups, depending on whether they were 20 years of age and older or under 20 years of age. Eighty‐eight patients under the age of 20 years who received human papillomavirus vaccination were excluded from the study. We reviewed clinical survey data and summaries of 200 Japanese patients with acquired autoimmune dysautonomia (AAD) who were seropositive for gAChR antibodies (mean age: 56.2 ± 21.4 years; 115 men and 85 women). Five patients were excluded from the group of adults because they developed AAD prior to the age of 20 years. We compared the clinical characteristics of children and adolescents with AAD and adults with AAD, seeking to determine the clinical features of pediatric AAD cases with seropositivity for gAChR antibodies in Japan.

We retrospectively reviewed the clinical information of the patients of AAD with gAChR Abs in serum who were treated in our clinic and were referred to our laboratory for antibody testing from January 27, 2012 to April 2, 2019 in Japan (Fig. 1). In this study, AAG, POTS, and AGID were targeted as immune‐mediated AAD, and the following diagnostic criteria were used. The diagnosis of AAG was confirmed using clinical criteria including frank orthostatic hypotension, gastrointestinal dysmotility, and seropositivity for anti‐gAChR autoantibodies.^20^, ^21^ The diagnosis of POTS was confirmed using the consensus statement by the American Autonomic Society, the European Federation of Autonomic Societies, the Autonomic Research Group of the World Federation of Neurology and the Autonomic Disorders section of the American Academy of Neurology, and the 2015 Heart Rhythm Society expert consensus statement.^22^, ^23^, ^24^ Clinical criteria were set for AGID requiring gAChR antibody positivity and some abdominal symptoms due to gastrointestinal dysmotility.^25^, ^26^ Clinical diagnoses were uniform at each hospital. All hospitals followed the same diagnostic guidelines described hereafter. Patients with an involvement of at least one autonomic domain and antibody positivity were considered to have AAD with gAChR Abs. Patients with alternative causes of autonomic dysfunction were excluded from the study.^27^, ^28^ The clinical diagnosis was confirmed with the diagnostic criteria and achieved following a clinical assessment by experienced neurologists, pediatricians, and gastroenterologists. Specific questionnaires and consent forms were sent to the referring neurologists, and the data were sorted and analyzed in Kumamoto, Japan. Data analysis was performed from October 3, 2017, to April 27, 2020.

Eighty‐eight patients under the age of 20 years who received human papillomavirus vaccination were also excluded (Fig. 1). These patients were excluded from the study because they are not directly related to the subject.^29^, ^30^ Finally, we reviewed the clinical survey data and summaries of 200 Japanese patients of AAD with gAChR Abs in serum (mean age: 56.2 ± 21.4 years; 115 men and 85 women), which were collected along with pre‐immunotherapy serum samples. Serum samples were centrifuged at 1006 *g* for 10 min and stored in cryovial tubes at −80°C within 2 h of collection. The samples were later sent to the Nagasaki Kawatana Medical Center or Kumamoto University Hospital.

### Clinical features

Comprehensive clinical assessments were performed for all patients of AAD with gAChR Abs in serum. Our questionnaire consisted of queries focused on the following topics: (1) age, sex, age at disease onset, disease duration, antecedent infection, and autonomic symptoms at onset; (2) autonomic manifestations described below; (3) extra‐autonomic manifestations (e.g., sensory disturbance, other neurological findings, and the presence of comorbid diseases including endocrine disorders, autoimmune diseases, and tumors).^27^, ^28^ Moreover, we confirmed the current history of autonomic symptoms appearing between several days and 2 weeks after antecedent infectious episodes.

We reviewed the clinical survey data and summaries of all patients and assessed the presence of the following symptoms that would indicate dysfunction of the autonomic system: orthostatic hypotension (OH) or orthostatic intolerance (OI), arrhythmia, pupillary dysfunction, coughing episodes, dryness of the skin, hypohidrosis or anhidrosis associated with heat intolerance, appetite loss, nausea/vomiting, early satiety, postprandial abdominal pain, and gastroparesis associated with dysfunction of the upper gastrointestinal (GI) system, diarrhea or constipation, and paralytic ileus associated with dysfunction of the lower GI system, dysuria or urinary retention associated with bladder dysfunction, and sexual dysfunction.^27^, ^28^

### COMPASS‐31

The patients with AAG enrolled since April 2014 completed a self‐administered questionnaire. COMPASS, a shortened version of the Composite Autonomic Symptom Score that was designed to quantitatively assess autonomic symptoms.^31^ It has six subscale weighted scores in the following domains: orthostatic intolerance (4 items; range, 0–40), vasomotor (3 items; range, 0–5), secretomotor (4 items; range, 0–15), gastrointestinal (12 items; range, 0–25), bladder (3 items; range, 0–10), and pupillomotor (5 items; range, 0–5). COMPASS is weighted according to published scoring methods to yield a total score of 0–100, with a score of 100 representing the highest, most severe degree of the autonomic symptom burden. The mean ± standard deviation score in healthy control subjects for this questionnaire was reported to be 9.67 ± 8.1.^32^

In this study, 151 participants (children and adolescents: 13; adults: 136) completed the questionnaire in Japanese within 15 min. However, the questions related to the vasomotor and pupillomotor domains were excluded, because it is often difficult for the Japanese subjects to judge skin color changes on an individual basis, and it is not customary for middle‐aged and older people to wear sunglasses or tinted glasses in Japan. The total scores were calculated by the summation of the individual item scores, with a possible maximum score of 90.^6^

### Autoantibody detection

Previously, the use of LIPS to diagnose AAG based on the presence of IgG Abs against both the *α*3 and *β*4 gAChR subunits detected in patient serum samples had been established and reported.^3^ In this study, serum gAChR Abs from patients with any form of autonomic dysfunction were detected using the LIPS assay.^3^ The gAChR Abs were measured at the Nagasaki Kawatana Medical Center and Kumamoto University Hospital, as previously described.^3^, ^19^, ^33^, ^34^ To assess the diagnostic accuracy of the LIPS assay, the cut‐off points for all data collected in the previous study were confirmed.^3^, ^19^, ^33^, ^34^ Based on the anti‐gAChR*α*3 and *β*4 Abs data from the healthy control subjects, the cut‐off values were calculated as mean plus three standard deviations (SDs) from the mean.^3^, ^19^, ^33^, ^34^ In this study, antibody levels were expressed as an antibody index (AI), calculated as follows: AI = measured relative luminescence units (RLU) value for the serum sample/RLU cut‐off value. The normal value established in this study from healthy individuals corresponded to an AI of <1.0.

### Statistical analyses

Commercially available statistical software (SigmaPlot^®^; SPSS, Inc., Chicago, IL) was used to analyze the data. SigmaPlot^®^ was used for data analysis. Statistical analyses were performed to compare the prevalence of symptoms and associated data between pediatric patients and adult patients of AAD with gAChR Abs in serum, and each of the analyses was assumed to be independent. Normally distributed data in both groups of patients of AAD with gAChR Abs in serum were analyzed by the Student’s *t*‐test for the continuous variables (age, age at onset, disease duration, levels of gAChR Abs, and COMPASS). The Mann–Whitney *U* test was employed in cases where the frequencies of Abs and other patient data were not normally distributed. For the categorical variables, the Chi‐square test was applied. For all analyses, *P* < 0.05 was considered to reflect a statistically significant difference.

## Results

### General clinical features

In this study, we included 195 (97.5%) of the 200 patients; five patients in the adult AAD group were excluded (Fig. 1) as autonomic symptoms in these five patients were under the age of 20 years, and their history of autonomic dysfunction and present illness status remained uncertain. Eighty‐one (41.5%) of the 195 patients were women. The median age at symptom onset was 53.5 years, and the median disease duration was 3.8 years. Ten patients (5.1%) had autoimmune POTS, 6 (3.1%) had AGID, and 179 (91.8%) had AAG.

The clinical features of the patients are listed in Table 1. Antecedent infectious episodes were reported in 31 patients (15.9%). Autonomic manifestations varied; however, OH, OI, and lower GI tract dysfunction were frequently observed (70.8%, 82.1%, and 75.9%, respectively). Extra‐autonomic manifestations, including comorbid diseases and central nervous system (CNS) involvement, including changes in personality, cognitive impairment, parkinsonism, and ataxia, were detected in 65 patients (33.3%), whereas sensory disturbance was observed in 91 patients (46.7%). Thirty‐three patients (16.9%) exhibited the following endocrine disorders: hyponatremia (*n* = 11), syndrome of inappropriate antidiuretic hormone secretion (SIADH) (*n* = 8), amenorrhea (*n* = 8), and others. Fifty‐eight patients (29.7%) presented with other autoimmune diseases, and Sjögren’s syndrome (SS) and Hashimoto’s disease were confirmed in 22 and 13 patients, respectively. Tumors were observed in 21 patients (10.8%), of which four patients had ovarian tumors and lung cancer, respectively (Table 1).

**Table 1:** Clinical features of 195 patients of AAD with serum gAChR antibodies

Age (yr)	57.1 ± 21.3
Gender, female (%)	81/195 (41.5)
Age at onset (yr)	53.5 ± 21.3
Disease duration (yr)	3.8 ± 5.6
Antecedent infectious episodes (%)	31/195 (15.9)
Autonomic symptom at onset, GI symptoms (%)	35/195 (17.9)
Orthostatic hypotension (%)	138/195 (70.8)
Orthostatic intolerance (%)	160/195 (82.1)
POTS (%)	10/195 (5.1)
Arrhythmia (%)	32/195 (16.4)
Pupillary abnormalities (%)	41/195 (21.0)
Dry eye and/or dry mouth (%)	89/195 (45.6)
Coughing episodes (%)	26/195 (13.3)
Anhidrosis or heat intolerance or dry skin (%)	93/195 (47.7)
Upper GI dysfunction (%)	86/195 (44.1)
Lower GI dysfunction (%)	148/195 (75.9)
AGID (%)	6/195 (3.1)
Bladder dysfunction (%)	106/195 (54.4)
Sexual dysfunction (%)	32/114 (28.1)
CNS involvements (%)	65/195 (33.3)
Sensory disturbance (%)	91/195 (46.7)
Endocrine disorder (%)	33/195 (16.9)
Autoimmune disease (%)	58/195 (29.7)
Tumor (%)	21/195 (10.8)
gAChR*α*3 Abs (%)	130/200 (65.0)
gAChR*β*4 Abs (%)	18/200 (9.0)
gAChR*α*3 and *β*4 Abs (%)	52/200 (26.0

Abbreviation: AAD, acquired autoimmune dysautonomia; Abs, antibodies; AGID, autoimmune gastrointestinal dysmotility; CNS, central nervous system; gAChR, ganglionic nicotinic acetylcholine receptor; GI, gastrointestinal; POTS, postural orthostatic tachycardia syndrome

### Antibody findings

We identified a total of 195 patients diagnosed with AAD and with auto‐gAChR seropositivity, which were all enrolled in this retrospective study. More specifically, anti‐gAChR*α*3 Abs were detected in 130 patients (65.0%), and anti‐gAChR*β*4 Abs were detected in 18 patients (9.0%). Furthermore, 52 patients (26.0%) with AAD were positive for both gAChR*α*3 and gAChR*β*4 Abs (Table 1). On comparing the group of gAChR*β*4 Ab‐positive cases (i.e., only gAChR*β*4 Ab positive and double‐positive) and the group of gAChR*β*4 Ab‐negative cases (i.e., the gAChR*α*3 Ab‐positive), no statistical difference was found in the clinical features and COMPASS‐31 between the gAChR*α*3 Ab‐positive group, the gAChR*β*4 Ab‐positive, and double‐positive groups (Table S1).

### Comparison of clinical characteristics between children and adolescents with AAD and adults with AAD

We compared the clinical features of children and adolescents with AAD and adults with AAD. Clinically, two remarkable findings were observed. These were related to disease duration and history of antecedent infectious episodes. Disease duration in children and adolescents with AAD was significantly shorter than that in adults with AAD (*P* = 0.035, Table 2). We found that antecedent infectious episodes were more frequent in children and adolescents with AAD (*P* < 0.001, Table 2) than in adults with AAD. In terms of cardiovascular autonomic dysfunction, the frequency of OH was significantly lower in children and adolescents than in adults with AAD (*P* = 0.016, Table 2). We confirmed that four patients (4/20, 20.0%) in the group of children and adolescents met the POTS diagnostic criteria,^22^ with the incidence of POTS being significantly higher in children and adolescents than in adults with AAD (*P* = 0.008, Table 2). Although we found no statistical difference for the sicca complex, dry eye only was more frequent in children and adolescents than in adults with AAD (*P* = 0.040, Table 2). Upper GI dysfunction was more common, especially in children and adolescents with AAD; nausea and vomiting occurred in 60.0% (12/20) of children and adolescents, and 21.1% (37/175) of adults (*P* < 0.001, Table 2). Although lower GI dysfunction tended to be more frequent in children and adolescents than in adults with AAD, there was no statistically significant difference between both groups. However, diarrhea was observed at a significantly higher frequency in children and adolescents than in adults (*P* = 0.002, Table 2). In contrast, we found that bladder dysfunction was less frequent in children and adolescents than in adults with AAD (*P* = 0.038, Table 2).

**Table 2:** Comparison of characteristics and autonomic manifestations between children with AAD and adults with AAD.

Clinical feature	Children with AAD (*n* = 20)	Adults with AAD (*n* = 175)	*P* value
Age (yr)	14.5 ± 4.3	61.9 ± 16.5	<0.001*
Gender, female (%)	9/20 (45.0)	72/175 (41.1)	0.927
Age at onset (yr)	12.3 ± 4.3	58.2 ± 17.0	0.028*
Disease duration (yr)	2.2 ± 3.6	4.0 ± 5.8	0.035*
Antecedent infectious episodes (%)	10/20 (50.0)	21/175 (12.0)	<0.001*
Autonomic symptom at onset, GI symptoms (%)	7/20 (35.0)	28/175 (16.0)	0.073
Orthostatic hypotension (%)	9/20 (45.0)	129/175 (73.7)	0.016*
Orthostatic intolerance (%)	17/20 (85.0)	143/175 (81.7)	0.956
POTS (%)	4/20 (20.0)	6/175 (3.4)	0.008*
Arrhythmia (%)	2/20 (10.0)	30/175 (17.1)	0.618
Pupillary abnormalities (%)	6/20 (30.0)	35/175 (20.0)	0.453
Dry eye and/or dry mouth (%)	11/20 (55.0)	78/175 (44.6)	0.516
Dry eye only (%)	4/20 (20.0)	9/175 (5.1)	0.040*
Dry mouth only (%)	3/20 (15.0)	33/175 (18.8)	0.907
Both (%)	4/20 (20.0)	36/175 (20.6)	0.816
Coughing episodes (%)	4/20 (20.0)	22/175 (12.6)	0.563
Anhidrosis or heat intolerance or dry skin (%)	10/20 (50.0)	83/175 (47.4)	0.985
Hypohidrosis or anhidrosis (%)	7/20 (35.0)	51/175 (29.1)	0.776
Heat intolerance (%)	2/20 (10.0)	16/175 (9.1)	0.778
Dry skin (%)	3/20 (15.0)	31/175 (17.7)	0.994
Upper GI dysfunction (%)	13/20 (65.0)	73/175 (41.7)	0.080
Appetite loss (%)	3/20 (15.0)	35/175 (20.0)	0.813
Nausea and vomiting (%)	12/20 (60.0)	37/175 (21.1)	<0.001*
Early satiety (%)	1/20 (5.0)	24/175 (13.7)	0.452
Postprandial abdominal pain (%)	2/20 (10.0)	14/175 (8.0)	0.903
Gastroparesis (%)	1/20 (5.0)	5/175 (2.9)	0.875
Lower GI dysfunction (%)	18/20 (90.0)	130/175 (74.3)	0.200
Constipation (%)	8/20 (40.0)	93/175 (53.1)	0.380
Diarrhea (%)	7/20 (35.0)	16/175 (9.1)	0.002*
Paralytic ileus (%)	4/20 (20.0)	11/175 (6.3)	0.062
Mixed or alternating constipation and diarrhea (%)	3/20 (15.0)	19/175 (10.9)	0.856
AGID (%)	0/20 (0.0)	6/175 (3.4)	0.875
Bladder dysfunction (%)	6/20 (30.0)	100/175 (57.1)	0.038*
Sexual dysfunction (%)	1/11 (9.1)	31/103 (30.1)	0.262
gAChR*α*3 Abs (%)	16/20 (80.0)	113/175 (64.6)	0.258
gAChR*β*4 Abs (%)	3/20 (15.0)	14/175 (8.0)	0.527
gAChR*α*3 and *β*4 Abs (%)	1/20 (5.0)	48/175 (27.4)	0.055

*P*‐value from Chi‐square test.

Abbreviation: AAD, acquired autoimmune dysautonomia; Abs, antibodies; AGID, autoimmune gastrointestinal dysmotility; gAChR, ganglionic nicotinic acetylcholine receptor; GI, gastrointestinal; POTS, postural orthostatic tachycardia syndrome

* *P* < 0.05.

With regard to extra‐autonomic manifestations, although CNS involvement, sensory disturbance, and endocrine disorders tended to be higher in children and adolescents with AAD, there was no statistically significant difference (Table 3). More specifically, encephalopathy was more frequent in children and adolescents (3/20, 15.0%) than in adults (2/175, 1.1%) (*P* = 0.003). Conversely, although the prevalence of autoimmune diseases and tumors was much higher in adults with AAD compared to children and adolescents, there was no statistically significant difference (Table 3).

**Table 3:** Comparison of extra‐autonomic manifestations between children with AAD and adults with AAD.

Clinical feature	Children with AAD (*n* = 20)	Adults with AAD (*n* = 175)	*P* value
CNS involvement (%)	8/20 (40.0)	57/175 (32.6)	0.638
Encephalopathy (%)	3/20 (15.0)	2/175 (1.1)	0.003*
Sensory disturbance (%)	11/20 (55.0)	80/175 (45.7)	0.581
Endocrine disorder (%)	5/20 (25.0)	28/175 (16.0)	0.483
Autoimmune disease (%)	3/20 (15.0)	55/175 (31.4)	0.206
Tumor (%)	0/20 (0.0)	21/175 (12.0)	0.208

*P*‐value from Chi‐square test.

Abbreviation: AAD, acquired autoimmune dysautonomia; CNS, central nervous system.

* *P* < 0.05.

In the group of children and adolescents with AAD, single seropositivity for the anti‐gAChR*α*3 Abs was observed in 16/20 patients (80.0%), whereas single seropositivity for the anti‐gAChR*β*4 Abs was observed in 3/20 patients (15.0%). One patient (5.0%) was positive for both Abs (Table 2). However, in the group of adults with AAD, single seropositivity for the anti‐gAChR*α*3 Abs was observed in 113/175 patients (64.6%), whereas single seropositivity for the anti‐gAChR*β*4 Abs was observed in 14/175 patients (8.0%). Forty‐eight patients (27.4%) were positive for both Abs. We failed to identify any statistically significant difference in these comparisons between both groups; however, double positives tended to be less in pediatric patients. (Table 2).

### Comparison of COMPASS‐31 between children and adolescents with AAD and adults with AAD

**Figure 2:**
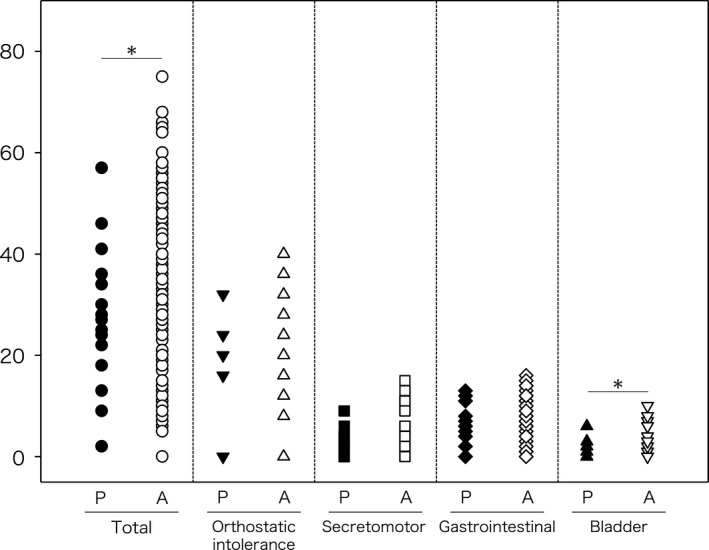
COMPASS‐31 in patients of AAD with gAChR Abs. We compared the COMPASS‐31 between the pediatric (P) and adult (A) patient populations of AAD with gAChR Abs. On comparing COMPASS‐31, total scores and bladder scores were significantly different between children and adolescents with AAD and adults with AAD (*P* = 0.035, *P* = 0.030, respectively).

The study confirmed the COMPASS data in 15/20 (75.0%) pediatric patients with AAD with gAChR Abs. The COMPASS results were as follows: median total score, 27.5 ± 14.3; median orthostatic intolerance score, 17.3 ± 10.3; median secretomotor score, 3.2 ± 3.0; median gastrointestinal score, 5.7 ± 4.2; median bladder score, 1.1 ± 1.7. Similar results were achieved with the COMPASS data in 136/175 (90.0%) adult patients with AAD who tested positive for gAChR Abs. The COMPASS yielded the following results: median total score, 37.1 ± 17.0; median orthostatic intolerance score, 22.1 ± 13.0; median secretomotor score, 4.8 ± 3.7; median gastrointestinal score, 7.4 ± 3.9; median bladder score, 2.7 ± 2.8. There was a significant difference in total score and bladder score in the comparison of COMPASS‐31 between children and adolescents with AAD and adults with AAD (*P* = 0.035, *P* = 0.030, respectively, Fig. 2).

## Discussion

The gAChR is expressed in the sympathetic, parasympathetic, and enteric ganglia in the peripheral autonomic ganglia. Hence, gAChR Abs have the potential to disrupt synaptic transmission in the autonomic ganglia and lead to autonomic failure.^35^ To the best of our knowledge, this study represents the first to describe the clinical characteristics of pediatric AAD patients who are positive for the gAChR Abs. AAD in children and adolescents is characterized clinically by short disease duration and the presence of such an antecedent infection as acute infectious disease. Since half of the pediatric patients with AAD showed progression following antecedent infections, immunological mechanisms similar to those of Guillain–Barré syndrome are suspected to represent the underlying pathological mechanism.

In this study, we confirmed that GI symptom onset, POTS, dry eye only, and nausea and vomiting among upper GI dysfunctions were frequent in pediatric patients with AAD. It remains unclear, however, why these symptoms were more prevalent in children and adolescents with AAD. In addition, there were 8 pediatric patients who had only OI without OH. Although these patients did not meet the diagnostic criteria for POTS, the tendency for POTS was inferred. Recently, the onset of POTS in teens has garnered significant attention, and many adolescents with POTS report symptoms of chronic fatigue and pain, with the most common complaints including headache, abdominal discomfort, and nonspecific generalized pain. In terms of abdominal discomfort, several studies pointed to a strong association between orthostatic changes and the pathogenesis of the GI symptoms.^36^, ^37^, ^38^ Sullivan et al. assessed a group of children with functional GI symptoms, mainly abdominal pain, nausea, and vomiting, who also underwent tilt table testing, and demonstrated an association with either POTS, neutrally mediated hypotension, or POTS with neutrally mediated hypotension.^38^ Moreover, the patients with POTS and GI symptoms had higher variability in terms of nerve activity frequency when assessed post‐prandially by electrogastrography.^39^ POTS and GI manifestations may be pathologically related in pediatric cases. Encephalopathy is also more frequently observed in children and adolescents than in adults. We previously reported a case of pediatric AAG presenting with acute encephalitis.^17^ However, it remains to be determined how child/adolescent cases are more likely to develop encephalopathy. Nicotinic AChRs are abundantly expressed throughout the central and peripheral nervous systems.^40^, ^41^ Nicotinic AChRs in the CNS are involved in higher brain function, including memory, cognition, and learning, among others.^42^ Given those facts, we speculate that nicotinic AChR*α*3 and *β*4 Abs may play a role in the development of encephalopathy in AAD.^17^, ^19^, ^27^, ^28^, ^43^, ^44^, ^45^, ^46^ It remains necessary to assess whether the development of the abovementioned clinical symptoms such as POTS, upper GI dysmotility, diarrhea, paralytic ileus, and encephalopathy is actually associated with the presence of anti‐gAChR Abs. In addition, it will be necessary to confirm whether pediatric AAD cases different clinical features from adult AAD cases due to their being in a developmental stage.

According to the attending pediatrician, children and adolescents with AAD have numerous complaints. Consequently, such children and adolescents may be diagnosed with orthostatic dysregulation disorders or psychosomatic disease, based on the abovementioned clinical features, making early diagnosis of AAD difficult. Although the severity of autonomic dysfunction seems to be milder in pediatric patients than in adult patients based on the comparison of COMPASS‐31 between these two groups, it is recommended that children undergo gAChR antibody testing if they present with symptoms such as POTS, functional GI manifestations, or neurological symptoms of unknown origins.

Our study has obvious limitations related to its retrospective design and the small pediatric patient sample size. A multicenter prospective study is essential to further analyze the clinical features of pediatric AAD and the efficacy of immunotherapy. In addition, although we assumed that each analysis was independent and did not require correction on repetition, the multiplicity of analysis may raise concerns. Despite these limitations, to the best of our knowledge, this study provides the most comprehensive report on the clinical characteristics of pediatric AAD cases to date.

## Conclusions

The results of this study and previous case reports have several practical implications. First, antecedent infections were more prevalent in pediatric AAD patients than in adult patients. Second, pediatric AAD patients demonstrate GI symptoms more often compared to adult AAD patients, including those with POTS and GI dysfunction. Third, encephalopathy, which is more frequent in pediatric AAD patients than in adult AAD patients, may be directly caused by gAChR Abs. These findings point to the heterogeneity of symptoms and clinical features in pediatric AAD cases.^47^ Fourth, we emphasize the importance of an accurate diagnosis of pediatric AAD with gAChR Abs and seamless transitional medical care from childhood to adulthood. Although we excluded patients in the adult AAD group, we should register the cases of pediatric AAD and adult AAD with childhood onset in the future. Finally, the findings of this study are important in terms of their implications with regard to how best to treat seronegative cases and to proceed with further research.

## Conflict of Interest

None of the authors have any conflicts of interest to disclose.

## Authors’ Contributions

Dr. Nakane had full access to all the data in the study and takes responsibility for the integrity of the data and the accuracy of the data analysis.

Conceived and designed the experiments: Yamakawa, Watari, Nakane; Performed the experiments: Yamakawa, Mukaino, Higuchi, Maeda; Acquisition, analysis, or interpretation of the data: All authors; Drafting of the manuscript and statistical analysis: Yamakawa, Watari, Nakane; Obtaining funding: Watari, Nakane, Study supervision: Nakane.

## Supporting information


**Table S1.** Comparison between the group of gAChR*α*3 Ab positive and the group of gAChR*β*4 Ab positive and double Ab positive.
