# Supplementary material for: gAChR antibodies in children and adolescents with acquired autoimmune dysautonomia in Japan
Source: Ann Clin Transl Neurol. 2021 Feb 23;8(4):790–9. doi: 10.1002/acn3.51317 (PMC8045944; doi:10.1002/acn3.51317)
Supplement: Supplementary file 1 — Table S1. Comparison between the group of gAChRα3 Ab positive and the group of gAChRβ4 Ab positive and double Ab positive. [file ACN3-8-790-s001.docx]

Supplementary data. Comparison between the group of gAChRα3 Ab positive and the group of gAChRβ4 Ab positive and double Ab positive

| Clinical features | gAChRα3 Abs positive only | gAChRβ4 Abs positive and double Abs positive | *P* value |
| --- | --- | --- | --- |
| n (COMPASS 31) | 130 (98) | 65 (53) |  |
| Gender, female (%) | 56/130 (43.1) | 25/65 (38.5) | 0.644 |
| Antecedent events (%) | 24/130 (18.5) | 10/65 (15.4) | 0.739 |
| Autonomic symptom at onset, OH or OI (%) | 78/130 (60.0) | 46/65 (70.8) | 0.188 |
| OH (%) | 87/130 (66.9) | 51/65 (78.5) | 0.133 |
| OI (%) | 106/130 (81.5) | 54/65 (83.1) | 0.947 |
| Arrhythmia (%) | 24/130 (18.5) | 8/65 (12.3) | 0.347 |
| Pupillary abnormalities (%) | 27/130 (20.8) | 14/65 (21.5) | 0.950 |
| Dry eye and/or dry mouth (%) | 58/130 (44.6) | 31/65 (47.7) | 0.799 |
| Coughing episodes (%) | 15/130 (11.5) | 11/65 (16.9) | 0.413 |
| Anhidrosis or heat intolerance or dry skin (%) | 58/130 (44.6) | 35/65 (53.8) | 0.287 |
| Upper GI dysfunction (%) | 62/130 (47.7) | 24/65 (36.9) | 0.202 |
| Lower GI dysfunction (%) | 99/130 (76.2) | 48/65 (73.8) | 0.860 |
| Bladder dysfunction (%) | 68/130 (52.3) | 39/65 (60.0) | 0.387 |
| Sexual dysfunction (%) | 20/74 (27.0) | 12/40 (30.0) | 0.905 |
| CNS involvement (%) | 43/130 (33.1) | 22/65 (33.8) | 0.957 |
| Sensory disturbance (%) | 57/130 (43.8) | 34/65 (52.3) | 0.335 |
| Endocrine disorder (%) | 24/130 (18.5) | 16/65 (24.6) | 0.415 |
| Autoimmune disease (%) | 37/130 (28.5) | 21/65 (32.3) | 0.698 |
| Tumor (%) | 12/130 (9.2) | 9/65 (13.8) | 0.462 |
| COMPASS: Total | 39.0 ± 16.3 | 34.6 ± 17.2 | 0.220 |
| COMPASS: OI | 23.6 ± 12.0 | 20.5 ± 13.2 | 0.196 |
| COMPASS: Secretomotor | 5.1 ± 3.7 | 4.4 ± 3.7 | 0.222 |
| COMPASS: GI | 7.6 ± 4.1 | 7.0 ± 3.9 | 0.460 |
| COMPASS: Bladder | 2.6 ± 2.7 | 2.6 ± 2.8 | 0.834 |

Ab, antibody; gAChR, ganglionic nicotinic acetylcholine receptor; CNS, central nervous system; COMPASS 31, composite autonomic symptom score 31; GI, gastrointestinal;

OH, orthostatic hypotension; OI, orthostatic intolerance
